# Supplementary material for: Early-life stress lastingly impacts microglial transcriptome and function under basal and immune-challenged conditions
Source: Transl Psychiatry. 2022 Dec 8;12:507. doi: 10.1038/s41398-022-02265-6 (PMC9731997; doi:10.1038/s41398-022-02265-6)
Supplement: Supplementary file 1 — Supplementary Materials [file 41398_2022_2265_MOESM1_ESM.docx]

Supplementary Figure Legends

Figure S1: ELS validation across cohorts used (related to Materials and Methods: ELS model), and morphological analysis of ELS and control microglia in adulthood under basal and immune-challenged conditions (related to figure 1).

(A) ELS decreases body weight gain from postnatal days 2-9 across various cohorts in this project. Body weight at P2 is based on the average body weight, by sex, in each nest. Two-tailed student’s t-test, *: main effect condition, p<0.05. (B, C) Iba1+ cell density (B) and coverage (C) in the dentate gyrus and cornu ammonis of the hippocampus. Two-tailed two-way ANOVA, *: main effect LPS, p<0.05; $: trend for LPS effect, p=0.053. (D) Effects of condition (CTR/ELS) and treatment (PBS/LPS) on the proportion of morphological microglia subtypes in the stratum lacunosum-moleculare of 3-5 months old CTR and ELS-exposed mice. General Linear Model Multivariate test, *: main effect treatment, #: treatment effect for subtype 1, 3 and 4, ^: condition effect for subtype 1. p<0.05. Abbreviations: CTR= control, ELS= early-life stress, ANOVA = analysis of variance, LPS = lipopolysaccharide, PBS = phosphate-buffered saline. Center values in bar graphs represent the mean, error bars represent the SEM. Stacked bar plot depicts the average proportion of each cell type per group.

Figure S2: Transcriptional analysis of ELS and control microglia in adulthood under basal and immune-challenged conditions (related to figure 1).

(A) FACS strategy to obtain single, viable (DAPI-, DRAQ5+), CD45+, CD11b+ microglia. (B) Average expression (logCPM) of gene sets specific for different brain cell types (64) (Table S2) in microglia of all experimental groups. (C) Pearson correlation (R2) of the first 6 principal components (PC) to the experimental variables (treatment (PBS/LPS), condition (CTR/ELS) and age (P9/P200)) (FDR ***<0.001, **<0.01, *<0.05). (D) Volcano plot depicting differential expressed genes between P9: ELS and P9: CTR (logFC</>1, FDR<0.05). Each dot represents a gene. The only significantly differentially expressed gene, Trem1, is labelled and marked with cyan. (E) Gene expression (CPM) of Trem1 in P9: ELS and P9: CTR microglia from individual mice. (F) Gene dendrogram and module colors of weighted gene co-expression network analysis. Abbreviations: AST = astrocytes, CTR = control, CPM = counts per million, ELS = early- life stress, END= endothelial cells, FC = fold change, FSC = forward scatter, LPS = lipopolysaccharide, MIC = microglia, NEU = neurons, OLI = oligodendrocytes, P = postnatal day, PC = principal component, PBS = phosphate-buffered saline, SSC = side scatter.

Figure S3: Plasma cytokines and cluster analysis of LPS-responsive genes in CTR and ELS microglia.

(A) Plasma cytokines from cohort 3; CTR and ELS mice injected with PBS or LPS (1 mg/kg), measured 3 hours post injection at P200 with a cytometric bead array. Two-tailed two-way ANOVA * main effect treatment (PBS/LPS), p< 0.0001 (B) Heatmap with Manhattan distance-based hierarchical clustering analysis depicting z-scores of average logCPM values of all genes overlapping between P200: CTR-LPS vs P200: CTR-PBS and P200: ELS-LPS vs P200: ELS-PBS. (related to figure 3). Abbreviations: CTR= control, ELS= early-life stress, LPS= lipopolysaccharide, PBS= phosphate-buffered saline.

Figure S4: Flow cytometry analysis of synaptosomal phagocytosis ex vivo (related to figure 4)

Gating strategy used in the ex vivo phagocytosis experiments. Live (DAPI-) cells were selected from the doublet population. The amount of phagocytosis was quantified as the proportion of phRodo+ CD11b+ cells divided by the total amount of live CD11b+ cells.

Supplementary tables

- Table S1. Clinical information from individuals used in the post-mortem study (related to Materials and Methods: Human cohort and Figure 5)**.**
- Table S2. publicly available gene lists of CNS cell types (64) and trained genes (53) doi: 10.1186/s40478-015-0203-5) (related to Figure S2B and Fig 3F).
- Table S3. Differential gene expression analysis P9: ELS vs. P9: CTR (logFC</>1, FDR<0.05) (related to Figure S2D,E).
- Table S4. Differential gene expression analysis P200: ELS-PBS vs. P200: CTR-PBS (logFC</>1, FDR<0.05) (related to Figure 1C).
- Table S5. Gene ontology analysis of genes identified in P200: ELS-PBS vs. P200: CTR-PBS microglia (p value<0.05) (related to Figure 1D).
- Table S6. Genes belonging to the pink module identified in WGCNA (related to Figure 1E, Figure S2F).
- Table S7. Gene ontology analysis of pink module genes (adjusted p value<0.05) (related to Figure 1F)
- Table S8. Differential gene expression analysis P200: CTR-PBS vs. P9: CTR (logFC</>1, FDR<0.05) (related to Figure 2A).
- Table S9. Differential gene expression analysis P200: ELS-PBS vs. P9: ELS (logFC</>1, FDR<0.05) (related to Figure 2A).
- Table S10. Original (enrichR) and reduced (rrvgo) gene ontology analysis of genes enriched in ELS and CTR microglia in the P200 vs P9 comparison (related to Figure 2C).
- Table S11. Original (enrichR) and reduced (rrvgo) gene ontology analysis of genes depleted in ELS and CTR microglia in the P200 vs P9 comparison (related to Figure 2D).
- Table S12. Differential gene expression analysis P200: CTR-LPS vs. P200: CTR-PBS (logFC</>1, FDR<0.05) (related to Figure 3A).
- Table S13. Differential gene expression analysis P200: ELS-LPS vs. P200: ELS-PBS (logFC</>1, FDR<0.05) (related to Figure 3A).
- Table S14. Original (enrichR) and reduced (rrvgo) gene ontology analysis of genes enriched in ELS and CTR microglia in the LPS vs PBS comparison (related to Figure 3B).
- Table S15. Original (enrichR) and reduced (rrvgo) gene ontology analysis of genes depleted in ELS and CTR microglia in the LPS vs PBS comparison (related to Figure 3C).
- Table S16. Clustering analysis of overlapping genes between the LPS response of ELS and CTR microglia (related to figure 3F).

**Supplementary methods**

**Breeding and early-life stress model**

Male (6 weeks of age) and primiparous female (8 weeks of age) C57Bl/6J mice were purchased from Harlan Laboratories B.V. (Venray, The Netherlands), housed in a controlled environment (temperature 22±1°C, humidity 55±5%) with *ad libitum* standard rodent chow and water, under a 12:12 h light-dark cycle schedule (lights on at 8 AM). After two weeks of acclimatization, mice were bred in house by housing two females with one male for one week. After another week of paired-housing of the two females, females were housed individually in a standard cage (type 1 short cage) covered with a filter top. Females were monitored daily, between 9 and 10 AM, for the birth of pups. When a litter was detected, the previous day was designated the day of birth (postnatal day (P)0). At P2, litters were randomly assigned to control (CTR) or early-life stress (ELS) groups. Only male mice were used in this study.

Early-life stress was induced via the limited bedding and nesting (LBN) stress paradigm as described before by our group and others (1–4). The LBN model induces fragmentation of maternal care resulting in chronic stress in the pups. At P2, litters were culled to six pups per litter (sex ratio male:female of 3:3 or 4:2) without cross fostering, randomly assigned to CTR or ELS condition. In ELS cages, the bottom was covered with a small amount of sawdust bedding and a fine-gauge stainless steel mesh was placed 1 cm above the cage floor. Half a square piece of cotton nesting material (2,5 x 5 cm, Technilab-BMI, Someren, the Netherlands) was placed on top of the mesh. Control cages were equipped with standard amounts of sawdust bedding and nesting material (one square piece of cotton nesting material (5 x 5cm). Animals were supplied with food and water *ad libitum* and covered with a filtertop. Throughout all procedures, manipulation was kept to a minimum to avoid handling effects and animals were left undisturbed until P9. All experiments and analyses were performed by researchers blinded to experimental conditions, with the exception of phagocytosis assays performed at P9, due to the visually distinct nature of the ELS treatment.

**Mouse tissue preparation**

For the immunohistochemistry cohort, mice were i.p. injected with 120 mg/kg Euthasol, then transcardially perfused with ice-cold PBS and 4% paraformaldehyde (PFA). Brains were post-fixed in 4 % PFA at 4˚C overnight before transfer to phosphate buffer (PB) with 0.01 % Na-Azide. Coronal brain sections (40 µm thick) were obtained using a sliding microtome after cryoprotection in 15% and 30% sucrose in 0.1 M PB. Sections were stored in antifreeze at -20˚C until use. For the transcriptomics cohort, mice were i.p. injected with 120 mg/kg Euthasol, and transcardially perfused with ice-cold PBS before dissecting out the hippocampus. For the synaptosome isolation and phagocytosis cohort, mice were sacrificed via rapid decapitation, with the respective brain tissues dissected out at 4˚C.

**Microglia staining**

Sections were incubated in 0.3 % H_2_O_2_ for 15 min to block endogenous peroxidase activity, then blocked in a ﻿0.05 M tris-buffered saline (TBS) solution containing 1% bovine serum albumin and 0.1% triton X-100 (pH 7.6) for 30 min. Samples were incubated in blocking mix containing primary antibody (1:5000 rabbit anti-IBA1, 019-19741, Wako) and incubated for 1 h at RT, then overnight at 4 degrees. The next day, sections were incubated for 2 h with 1:500 goat anti-rabbit biotinylated (Vector Laboratories) in blocking mix, after which sections were incubated with 1:800 avidin-biotin complex (Vectastain elite ABC-peroxidase kit, Brunschwig Chemie) in 0.05 M TBS. Finally, sections were washed in 0.05 TB (pH 7.6), incubated in 0.2 mg/1 ml diaminobenzidine (DAB, 0.01% H2O2 in 0.05 M TB) for the chromogen development, then mounted on pre-coated glass slides (Superfrost Plus slides, Menzel) and cover slipped using Entellan^TM^. Sections were washed in 0.05M Tris buffered saline in between steps unless otherwise specified.

Microglia subtype classification:

We subdivided microglia in a 20.000 µm^2^ region of interest within the hilus of the dentate gyrus and the stratum lacunosum-moleculare of the cornu ammonis as follows: type 1, ramified cells with many long, thin processes; type 2, cells with a clear distinguished soma with thicker longer processes; type 3, spider-like cells with no clear distinguished soma and numerous stout processes; type 4, cells with less than three short, thick processes; type 5, round, amoeboid cells without visible processes (Figure 1B, S1E).

**Microglia isolation**

After saline perfusion, hippocampi were dissected and collected in HBSS (Gibco, 14170-088) containing 15 mM HEPES (Lonza, BE17-737E), termed medium A, and 0.6 % glucose (Sigma-Aldrich, G8769)) at 4°C. Microglia were isolated on ice as described elsewhere (5). Briefly, a single cell suspension was obtained through mechanical dissociation of the brain tissue in a glass homogenizer filled with medium A. The cell suspension was filtered with a 70 µm cell strainer (Falcon, 352350). For brain tissue of adult mice, myelin and other lipids were removed by Percoll (Cytiva, 17-5445-02) gradient centrifugation at 950 xg for 20 min with brakes off. Percoll was diluted 9:1 in 10x HBSS (Gibco, 14180-046) and was regarded as 100% Percoll, which was further diluted in Medium A to 24.4 % Percoll. After this centrifugation step, cells were resuspended in colorless medium A (HBSS (Gibco, 14170-053) containing 15 mM HEPES, 0.6% glucose, and 1 mM EDTA (Invitrogen, 15575-038). Since myelin is nearly absent in the postnatal brain, Percoll gradient centrifugation was not performed for brain tissue of P9 mice. Unspecific binding of antibodies to the cells was prevented by incubating the cells for 15 minutes on ice with anti-mouse Fc receptor (5 µg/ml, eBioscience, 14-0161). Following the cells were stained with anti-mouse CD11b-PE (1.2 µg/ml, eBioscience, 12-0112) and anti-mouse CD45-FITC (2.5 µg/ml, eBioscience, 11-0451). Shortly before cell sorting DAPI (0.15 µg/ml, Biolegend, 422801) and DRAQ5 (2 µM, Thermo Scientific, 62251) were added to the cell suspension. Single, viable (DAPI^-^, DRAQ5^+^) microglia (CD45^int^, CD11b^+^) were sorted with the Beckman Coulter MoFlo XDP and were collected in 350 µl RNA lysis buffer (Qiagen, 1053393). Lysates were stored at -80°C until further use.

RNA isolation and mRNA sequencing

RNA was isolated with the RNeasy Plus Micro RNA isolation kit following the manufacturer’s protocol (Qiagen, 74034). The RNA concentration was determined with the Agilent tapestation system and the HS RNA kit. Library preparation, sequencing and primary data analysis were performed at GenomeScan in Leiden, the Netherlands. The NEBNext Low Input RNA Library Prep Kit for Illumina was used to process the samples. The sample preparation was performed according to the protocol "NEBNext Low Input RNA Library Prep Kit for Illumina" (NEB #E6420S/L). Briefly, cDNA was synthesized and amplified from poly-A-tailed mRNA. This was used for ligation with the sequencing adapters and PCR amplification of the resulting product. The quality and yield after sample preparation was measured with the Fragment Analyzer. The size of the resulting products was consistent with the expected size distribution (a peak between 200-400 bp). Clustering and DNA sequencing using the NovaSeq6000 was performed according to manufacturer's protocols. A concentration of 1.1 nM of DNA was used. The NovaSeq control software NCS v1.7 was used.

mRNA sequencing data analysis

Image analysis, base calling, and quality check was performed with the Illumina data analysis pipeline RTA3.4.4 and Bcl2fastq v2.20. Raw sequencing reads may contain low-quality, adaptor-polluted, or high content of unknown base reads. These should be processed to be removed before downstream analyzes. Sequence reads were trimmed to remove possible adapter sequences using cutadapt v2.10. Presumed adapter sequences were removed from the read when the bases matched a sequence in the adapter sequence set (TruSeq adapters).

For each sample, the trimmed reads were mapped to the mouse genome GRCm39 (Mus_musculus.GRCm39.dna.primary_assembly.fa). The reads were mapped to the reference sequence using a short-read aligner based on Burrows—Wheeler Transform (STAR2 v2.5.4) with default settings. The aligner is a splice-aware mapper that detects splice junctions and incorporates them to help align the entire read. Based on the mapped locations in the alignment file the frequency of how often a read was mapped on a transcript was determined with HTSeq v0.11.0. The hit counts were summarized and reported using the gene_id feature in the annotation file. Only unique reads that fall within exonic regions were counted. The counts were saved to count files, which served as input for downstream mRNA-seq analysis.

Differential gene expression analysis (±logFC >1, FDR<0.05) was done using a generalized linear model in edgeR (v3.32.1)(6) after exclusion of lowly expressed genes (total count < 1 in more than 2 samples) and normalization using trimmed mean of M-values.

A signed weighted gene co-expression analysis was done only on the P200 samples using the WGCNA package (v1.70-3)(7) on genes (total count >1000) VST-transformed with DeSeq2 (v1.24.0)(8). The adjacency and dissimilarity (1-dissTOM) matrix were calculated with a soft power threshold of 12. Gene modules were identified by hierarchical clustering with a minimal size of 150 genes. Metadata variables (condition, treatment) were converted to binary variables and were correlated to the previously identified module eigengenes, with p-value<0.05 being considered significant.

Gene ontology (GO) analysis for differentially expressed genes and gene modules was performed with enrichR (v3.0)(9) based on the "GO_Biological_Process_2021" database. Enrichment was based on adjusted p-value<0.05. When no GO terms were enriched with an adjusted p-value<0.05, GO terms with a p-value<0.05 were reported. GO terms shown in Figure 2 and 3 were reduced into parent terms using a semantic similarity matrix using the rrvgo package (v1.2.0)(10). The sum of total gene counts of all GO terms within one parent term was calculated and reported. Certain GO terms are reported in-text with their associated genes; for terms with >4 associated genes, the top 5 are listed based on absolute logFC.

Heatmap clustering was performed using the ward. D2 clustering method and Manhattan distance as clustering metrics, and visualized with pheatmap (v1.0.12) (11). Calculation of Pearson correlation between the first six principal components (PC) and experimental variables (age, condition, treatment), was done with the eigencorplot function of PCAtools (v2.2.0)(12), correcting for multiple testing using the Benjamini-Hochberg procedure. The first six PCs were used since they were identified with the Elbow method and Horn’s parallel analysis to explain the most variance in our data. Venn diagrams were made with VennDiagram (v1.6.20)(13). Alluvial plots were generated with ggalluvial (v0.12.3) (14). All other plots were generated with ggplot2 (v3.3.3.9000)(15).

**Hippocampal synaptosome isolation and conjugation**

Hippocampi from P9 or P150 mice were dissected and stored on dry ice until synaptosome isolation based on a published protocol (16). Hippocampi were homogenized in a buffer (0.32 M sucrose + 5 mM HEPES in 1x DPBS pH=7.4, with protease inhibitor cocktail Roche, 04693159001) using a tight Dounce pestle and centrifuged at 1000g for 10min at 4˚C. The supernatant was overlaid on a discontinuous 0.85M/1.2M sucrose gradient and ultracentrifuged at 100,000g for 2 h. The 0.85M/1.2M interphase was collected and diluted with more homogenization buffer, and the synaptosomes were precipitated out by ultracentrifugation 76,000g for 30 min. Synaptosomes were resuspended in 100µl 0.1M NaHCO_3_, and their concentrations determined via BCA Assay (Thermo Scientific, 23225). Synaptosomes were conjugated to phRodo-red (P36600, Invitrogen) according to manufacturer instructions, and diluted to a concentration of 7.5µg/µl.

**Microglia enrichment for ex vivo phagocytosis assay**

After sacrifice via rapid decapitation, whole brains (pups) or half brains from the cortex until the midbrain (adults) were collected into medium A, consisting of 1x HBSS (Gibco, 14170-088) with 15mM HEPES (Lonza, BE17-737E), and 0.6 % glucose (Sigma-Aldrich, G8769). The brains were homogenized using a loose Dounce pestle on ice, before passing through a pre-wet 70 µm filter. After a 6 min centrifugation at 500 x*g* and 4˚C, pellets were resuspended in 70 % isotonic Percoll (Cytiva, 17-5445-02) solution, which was overlaid with either 30% (pups) or 37% (adults) isotonic Percoll and centrifuged cold for 900 x*g* at 25min (pups), or 2000 *x*g at 20min (adults). 2ml of the resulting interphase was collected, diluted with 10 ml (pups) or 6 ml (adults) medium A, and centrifuged. The pellet was resuspended in 200µl of DMEM-F12 solution, counted using an automated cell counter (Bio-rad), and seeded into polystyrene FACS tubes at a concentration of 50,000 (pups) or 80,000 (adults) cells in 300µl DMEM-F12.

Cells were incubated with 1.25µg (pups) or 2µg (adults) phRodo-conjugated synaptosomes from age-matched CTL or ES mice for 30min, after which they were washed with 600µl DMEM/F12 and centrifuged. The pellet was washed again with FACS buffer (1x DBPS with 0.5% BSA and 2mM EDTA), before blocking in a 1:100 CD16/CD32 (Thermofisher 14-0161-82) in FACS buffer solution for 15 min. Cells were then stained 1:100 using APC-conjugated CD11b (Thermofisher, 17-0112-82) for 30min. Cells were then washed with 1ml FACS buffer, centrifuged, and resuspended before flow cytometry analysis.

**Human cohort and Fluorescent *in situ* hybridization in post-mortem human hippocampus**

Fresh-frozen hippocampal blocks from well-characterized age-matched adult males (depressed suicides with a history of child abuse and matched sudden-death controls) were obtained from the Douglas-Bell Canada Brain Bank (Montreal, Canada). Phenotypic information was retrieved through standardized psychological autopsies, in collaboration with the Quebec Coroner’s Office and with informed consent from next of kin. Presence of any or suspected neurological/neurodegenerative disorder signaled in clinical files constituted an exclusion criterion. Cases and controls are defined with the support of medical charts and Coroner records. Proxy-based interviews with one or more informants best acquainted with the deceased are supplemented with information from archival material obtained from hospitals, Coroner’s office, and social services. Clinical vignettes are then produced and assessed by a panel of clinicians to generate Diagnostic and Statistical Manual of Mental Disorders (DSM-IV) diagnostic criteria, providing sociodemographic characteristics, social developmental history, DSM-IV axis I diagnostic information, and behavioral traits—information that is obtained through different adapted questionnaires. Toxicological assessments and medication prescription are also obtained. As described previously, characterization of early-life histories was based on adapted Childhood Experience of Care and Abuse interviews assessing experiences of sexual and physical abuse, as well as neglect, and for which scores from siblings are highly concordant. We considered as severe early-life adversity (ELA) reports of non-random major physical and/or sexual abuse during childhood (up to 15 years). Only cases with the maximum severity ratings of 1 and 2 were included. This information was then complemented with medical charts and Coroner records. Because of this narrow selection criterion, it was not possible to stratify different types of abuse within the sample (17). Group characteristics can be found in Table S1, together with correlations between covariates (age, post-mortem interval (PMI), pH, substance dependence, and medication) and the variables measured in this study.

Hippocampal tissues were cut serially with a cryostat and 10 µm-thick sections were collected on Superfrost charged slides. *In situ* hybridization was performed using Advanced Cell Diagnostics RNAscope® probes and reagents (ACD Bio, 323110) following the manufacturer’s instructions. Sections were first fixed in cold 10% neutral buffered formalin for 15 min, dehydrated by increasing gradient of ethanol baths, and air dried for 5 min. Endogenous peroxidase activity was quenched with hydrogen peroxide for 10 min followed by protease digestion for 30 min at room temperature. The following probes were then hybridized for 2 h at 40 °C in a humidity-controlled oven: Hs-TMEM119 (ACD Bio 478911) and Hs-GAS6 (ACD Bio 455141). Amplifiers were added using the AMP reagents and the signal visualized through probe-specific HRP-based detection by tyramide signal amplification (TSA) with Opal dyes (Opal 520 and Opal 570; Perkin Elmer) diluted 1:900. To eliminate endogenous autofluorescence from lipofuscin and cellular debris, sections were incubated with TrueBlack (Biotium, 23007) for 30s. Slides were then coverslipped with Vectashield mounting medium with 4′,6-diamidino-2-phenylindole (DAPI) for nuclear staining (Vector Laboratories, H-1800) and kept at 4 °C until imaging. Both positive and negative controls provided by the supplier were used on separate sections to confirm signal specificity. Hippocampal sections were imaged using Olympus VS120 virtual slide microscope at 20x resolution. Dentate gyrus area was demarcated manually and QuPath (v0.3.2)(18) was employed for automated cell detection based on DAPI staining and RNAscope signal quantification. For stringent quantification, only cells bearing 3 or more fluorescence puncta for each probe were counted as positive.

**References**

1. Rice CJ, Sandman CA, Lenjavi MR, Baram TZ. A novel mouse model for acute and long-lasting consequences of early life stress. Endocrinology. 2008;149(10):4892–900.

2. Naninck EFG, Hoeijmakers L, Kakava-Georgiadou N, Meesters A, Lazic SE, Lucassen PJ, et al. Chronic early life stress alters developmental and adult neurogenesis and impairs cognitive function in mice. Hippocampus. 2015;25(3):309–28.

3. Hoeijmakers L, Ruigrok SR, Amelianchik A, Ivan D, van Dam AMM, Lucassen PJ, et al. Early-life stress lastingly alters the neuroinflammatory response to amyloid pathology in an Alzheimer’s disease mouse model. Brain Behav Immun. 2017;63:160–75.

4. Yam KY, Schipper L, Reemst K, Ruigrok SR, Abbink MR, Hoeijmakers L, et al. Increasing availability of ω-3 fatty acid in the early-life diet prevents the early-life stress-induced cognitive impairments without affecting metabolic alterations. FASEB J. 2019;33(4):5729–40.

5. Galatro TF, Vainchtein ID, Brouwer N, Boddeke EWGM, Eggen BJL. Isolation of microglia and immune infiltrates from mouse and primate central nervous system. In: Methods in Molecular Biology. 2017. p. 333–42.

6. Robinson MD, McCarthy DJ, Smyth GK. edgeR: A Bioconductor package for differential expression analysis of digital gene expression data. Bioinformatics. 2009;26(1):139–40.

7. Langfelder P, Horvath S. WGCNA: An R package for weighted correlation network analysis. BMC Bioinformatics. 2008;9(1):559.

8. Love MI, Huber W, Anders S. Moderated estimation of fold change and dispersion for RNA-seq data with DESeq2. Genome Biol. 2014;15(12):550.

9. Xie Z, Bailey A, Kuleshov M V., Clarke DJB, Evangelista JE, Jenkins SL, et al. Gene Set Knowledge Discovery with Enrichr. Curr Protoc. 2021;1(3):e90.

10. Sayols S. rrvgo: a Bioconductor package to reduce and visualize Gene Ontology terms. 2020;

11. Kolde R. pheatmap: Pretty Heatmaps. R package version 1.0.12. 2019;

12. Blighe, Kevin, and Lun A. PCAtools: everything Principal Components Analysis. 2019.

13. Chen H, Boutros PC. VennDiagram: A package for the generation of highly-customizable Venn and Euler diagrams in R. BMC Bioinformatics. 2011;12.

14. Brunson JC. ggalluvial: Alluvial Plots in “ggplot2”. 2020.

15. Wickham H. ggplot2: elegant graphics for data analysis. 2016.

16. Gonzalez-Lozano MA, Koopmans F, Sullivan PF, Protze J, Krause G, Verhage M, et al. Stitching the synapse: Cross-linking mass spectrometry into resolving synaptic protein interactions. Sci Adv. 2020;6(8).

17. Tanti A, Belliveau C, Nagy C, Maitra M, Denux F, Perlman K, et al. Child abuse associates with increased recruitment of perineuronal nets in the ventromedial prefrontal cortex: a possible implication of oligodendrocyte progenitor cells. Mol Psychiatry. 2021;27(3).

18. Bankhead P, Loughrey MB, Fernández JA, Dombrowski Y, McArt DG, Dunne PD, et al. QuPath: Open source software for digital pathology image analysis. Sci Rep. 2017;7(1).
