# Supplementary figures and images for: Early-life stress lastingly impacts microglial transcriptome and function under basal and immune-challenged conditions
Source: Transl Psychiatry. 2022 Dec 8;12:507. doi: 10.1038/s41398-022-02265-6 (PMC9731997; doi:10.1038/s41398-022-02265-6)

Figure S1

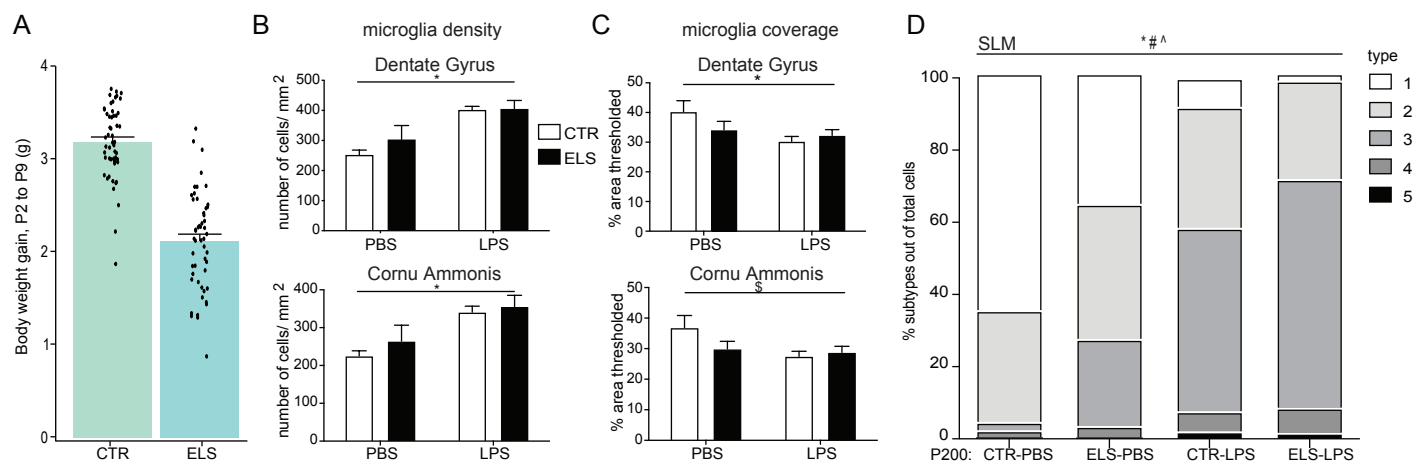

Supplement: Supplementary file 2 — Figure S1 [file 41398_2022_2265_MOESM2_ESM.pdf]

Figure S2

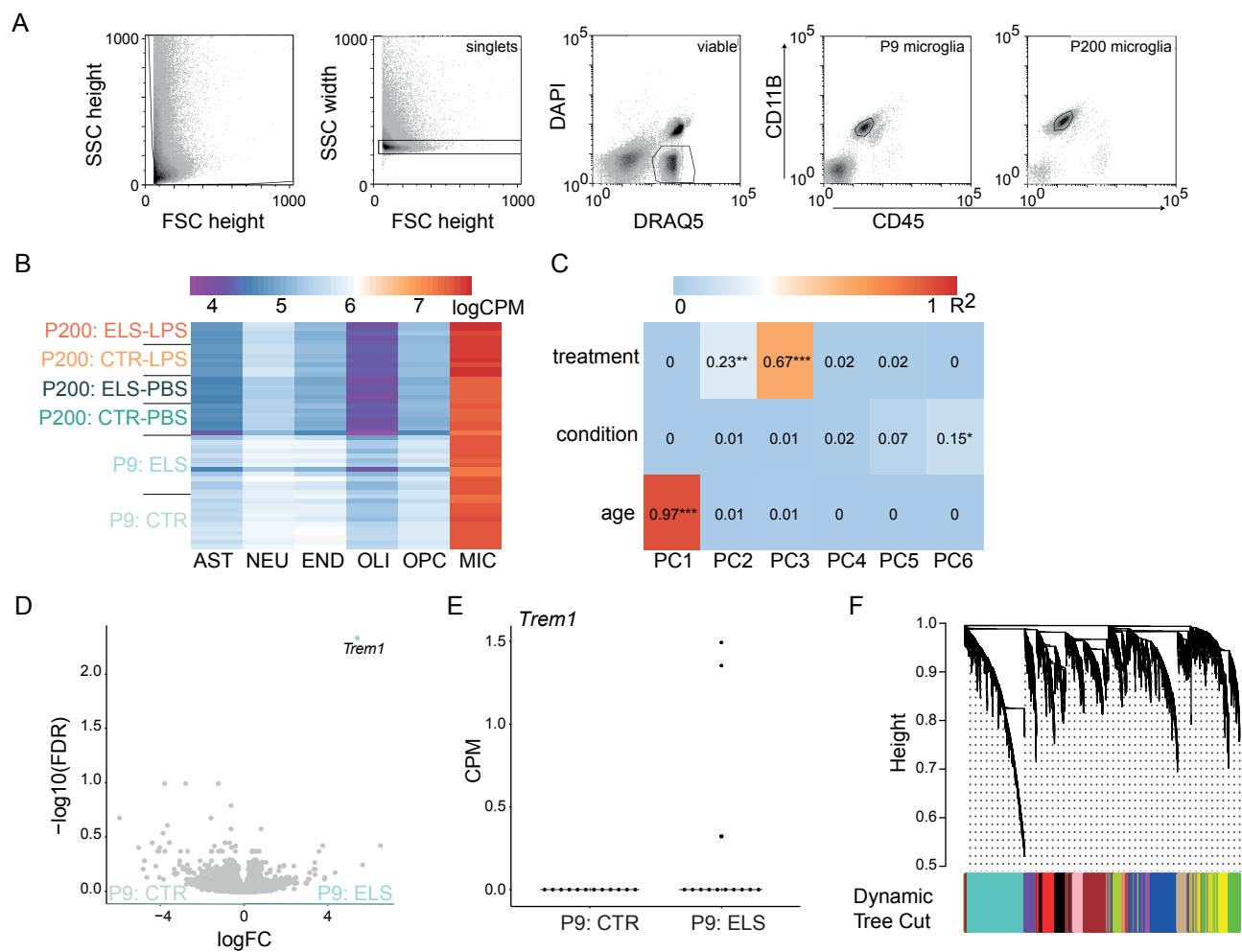

Supplement: Supplementary file 3 — Figure S2 [file 41398_2022_2265_MOESM3_ESM.pdf]

Figure S4

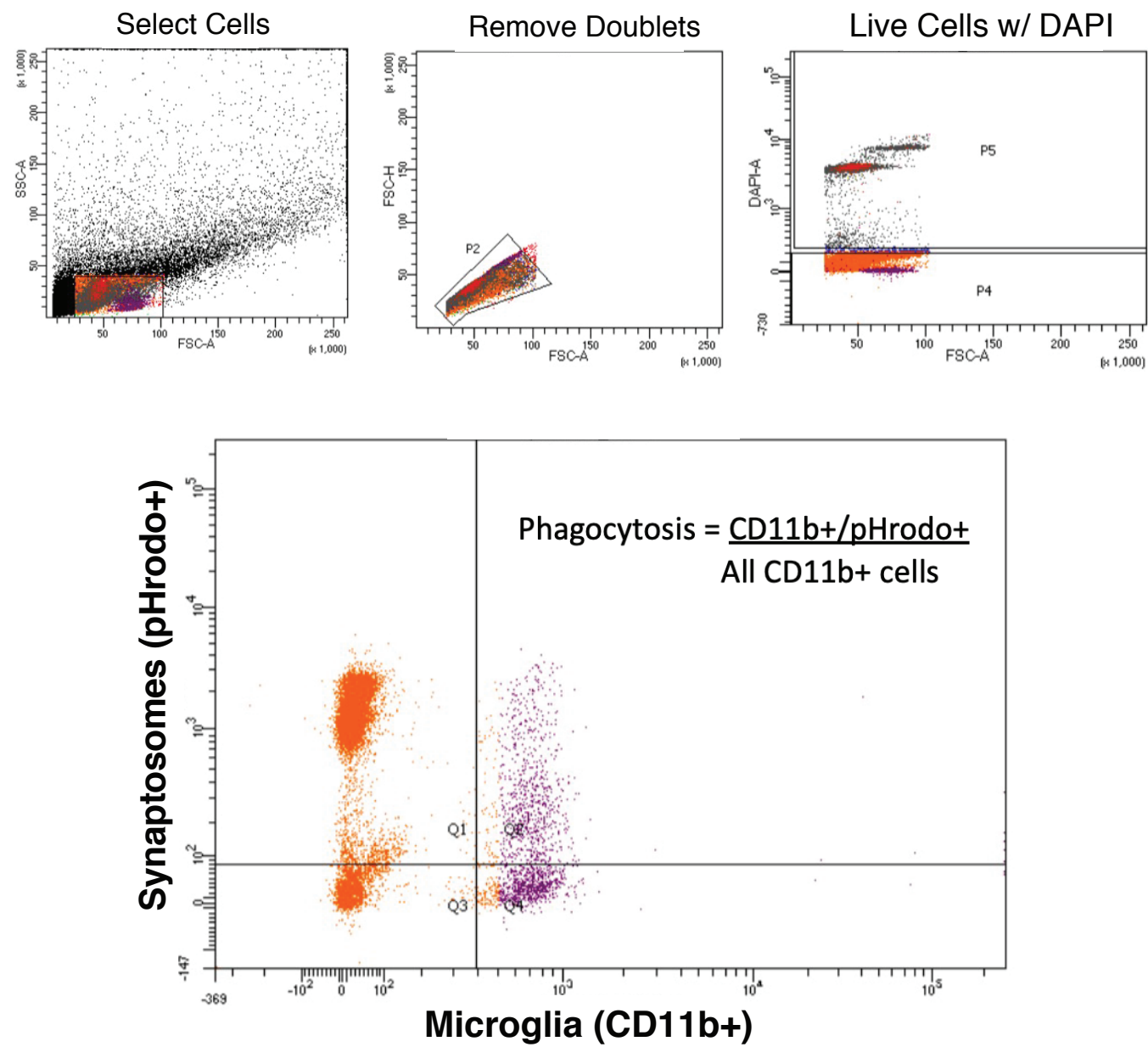

Supplement: Supplementary file 5 — Figure S4 [file 41398_2022_2265_MOESM5_ESM.pdf]
