# Supplementary material for: Early-life stress lastingly impacts microglial transcriptome and function under basal and immune-challenged conditions
Source: Transl Psychiatry. 2022 Dec 8;12:507. doi: 10.1038/s41398-022-02265-6 (PMC9731997; doi:10.1038/s41398-022-02265-6)

Figure S3

A

Experimental group	IFNγ Mean	IFNγ SD	IL6 Mean	IL6 SD	IL10 Mean	IL10 SD	IL12p70 Mean	IL12p70 SD	MCP-1 Mean	MCP-1 SD	TNF Mean	TNF SD
CTL-PBS (N=7)	0.669	0.062	23.806	3.507	88.454	15.591	16.925	5.769	26.024	4.384	0.766	0.403
ELS-PBS (N=6)	0.738	0.144	49.995	40.976	88.281	12.939	31.937	32.437	39.968	17.441	0.763	0.345
CTL-LPS (N=7)	4.295	1.533	14898.098	1432.613	569.667	234.826	41.470	11.660	5367.950	718.695	668.056	464.294
ELS-LPS (N=5)	3.986	1.264	14837.980	1805.400	606.816	159.516	61.950	63.274	5402.863	1398.141	651.438	570.128
**Two-way ANOVA**	**F1.21**	**p-value**	**F1.21**	**p-value**	**F1.21**	**p-value**	**F1.21**	**p-value**	**F1.21**	**p-value**	**F1.21**	**p-value**
Condition	0.449	0.510	2.422	0.135	0.008	0.931	1.557	0.226	0.570	0.459	0.003	0.954
Treatment	75.949	<0.0001*	1135.063	<0.0001*	74.435	<0.0001*	4.258	0.052	342.913	<0.0001*	21.318	0.0001*
Condition X Treatment	0.224	0.641	0.009	0.923	0.103	0.751	0.043	0.837	0.001	0.972	0.003	0.954

B

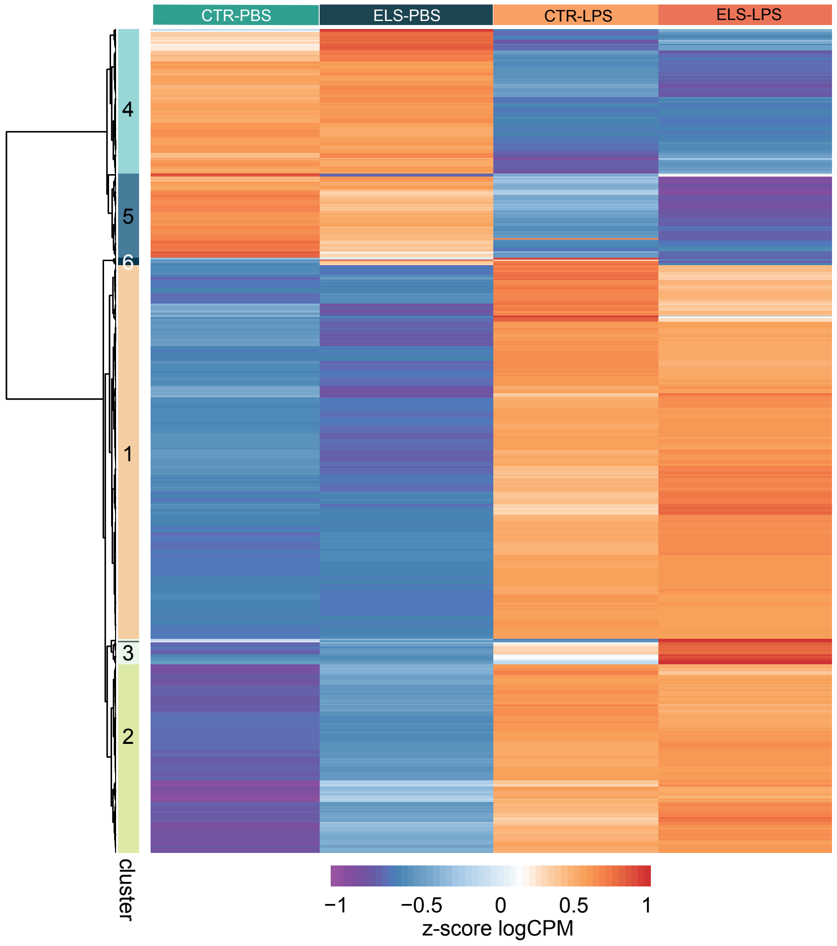

Supplement: Supplementary file 4 — Figure S3 [file 41398_2022_2265_MOESM4_ESM.pdf]
